# Cystic Fibrosis-Related Diabetes

**DOI:** 10.3389/fendo.2018.00020

**Published:** 2018-02-20

**Authors:** Kayani Kayani, Raihan Mohammed, Hasan Mohiaddin

**Affiliations:** ^1^Faculty of Medicine, University of Cambridge, Cambridge, United Kingdom; ^2^Faculty of Medicine, Imperial College London, London, United Kingdom

**Keywords:** diabetes, cystic fibrosis, cystic fibrosis transmembrane conductance regulator, pathophysiology, complications, treatment

## Abstract

Cystic fibrosis (CF) is the most common autosomal recessive disorder in Caucasian populations. Individuals with CF have seen significant increases in life expectancy in the last 60 years. As a result, previously rare complications are now coming to light. The most common of these is cystic fibrosis-related diabetes (CFRD), which affects 40–50% of CF adults. CFRD significantly impacts the pulmonary function and longevity of CF patients, yet a lack of consensus on the best methods to diagnose and treat CFRD remains. We begin by reviewing our understanding of the pathogenesis of CFRD, as emerging evidence shows the cystic fibrosis transmembrane conductance regulator (CFTR) also has important roles in the release of insulin and glucagon and in the protection of β cells from oxidative stress. We then discuss how current recommended methods of CFRD diagnosis are not appropriate, as continuous glucose monitoring becomes more effective, practical, and cost-effective. Finally, we evaluate emerging treatments which have narrowed the mortality gap within the CF patient group. In the future, pharmacological potentiators and correctors directly targeting CFTR show huge promise for both CFRD and the wider CF patient groups.

## Cystic Fibrosis (CF)

Cystic fibrosis is the most common lethal autosomal recessive disorder, affecting approximately 1 in 2,500 live births with a carrier frequency of 1/36 among northern Europeans (1). Almost 70,000 people live with CF globally (2). CF arises from mutations in the cystic fibrosis transmembrane conductance regulator (CFTR) gene that causes sufferers to experience thick, sticky mucous secretions in multiple mucin-producing organs. This gives rise to the characteristic lung pathology as well as problems in the gastrointestinal system, pancreas (exocrine and endocrine), reproductive system, and osteology (3). Since CFTR’s identification as the causative gene, extensive work has gone into understanding this gene and its roles in CF (4).

The CFTR gene is a cAMP-regulated chloride channel that is expressed in the apical membranes of various epithelial cells (5). The channel is part of the ATP-binding cassette transporter superfamily, meaning it hydrolyzes and conducts ATP. It also regulates vesicle trafficking and a number of apical membrane associated channels, including the epithelial sodium channel, the outwardly rectifying chloride channel, and the retinal outer medullary potassium channel (6, 7). Furthermore, CFTR has a role in regulating bicarbonate release, reducing the pH of secretions, especially in the pancreas, duodenum, ileum, and lung (8). Dysfunctional bicarbonate secretion is believed to be the major cause of CF pathology in these organs (9, 10).

Loss of CFTR function has been identified as disease causing. There are over 2000 mutations of the CFTR gene recorded in the Cystic Fibrosis Mutation Database (11). Of these, tests are available for 70, which will identify at least one mutant in most patients. However, in 18% of patients, only one abnormal allele is identified. CF mutations can be grouped as severe or mild and further categorized as one of six classes. Severe mutations are classes I–III, while mild mutations are classes IV–VI. Ultimately, these mutations affect CFTR in reaching, remaining, and/or functioning at the cell surface, impacting on secretory processes involving this channel, giving rise to CF pathology. For a review on CF mutational classes, see Wang et al. (12).

## Cystic Fibrosis-Related Diabetes (CFRD)

In the 1950s, CF patients had a life expectancy of less than a year. By 1970, it was 8 years. This low life span was multifactorial, but mostly attributable to poor management of pulmonary infections and to a lack of understanding of the defect in CF. Improvements in both have dramatically improved CF life expectancy. The CF Foundation patient registry (13) shows that the median predicted survival age in 2013 was 40.7 years in the USA population, compared to 45.1 years currently in the UK (14). Furthermore, a recent population based cohort study found that the median age of survival in Canada (based on 2009–2013 data) was 10 years greater than in the United States (50.9 vs. 40.6 years, respectively) (15). The authors suggested that differential access to transplantation related to the referral or donor lung allocation process; variable post-transplant survival; and differences in health care systems, including access to insurance, may in part explain the different rates in mortality.

However, this improvement in survival means patients are now experiencing complications in addition to lung disease and impaired nutrition. The most common of these is CFRD, with liver disease, bone disease, distal intestinal obstructive syndrome, gastroesophageal reflux disease, and depression also commonly observed (2). Two percent of CF patients have CFRD in childhood, increasing to 20% of adolescents, eventually reaching 40–50% of CF patients in adulthood (16). This expanding proportion suggests a multifactorial, complex, and progressive pathogenesis of CFRD.

Survival is significantly impacted in CFRD patients, with fewer than 25% surviving to an age of 30, compared to 60% of CF patients with normal glucose tolerance (NGT) (17). CFRD had a disproportionate impact on females, who showed a 16.3-year reduction in lifespan when compared to NGT female CF patients. By contrast, male CFRD patients only saw a 0.4-year reduction in longevity (18). Sims et al. (2005) (19) observed a greater reduction in lung forced expiratory volume in 1 s (FEV_1_) in female CFRD patients compared to NGT CF patients. This gender imbalance in CFRD is exacerbated by the higher prevalence of CFRD in females (20, 21). Although the cause for this sex difference in CFRD mortality was unclear; in 2009, Moran et al. (16) reported “previously noted sex differences in mortality have disappeared, and the gap in mortality between CF patients with and without diabetes has considerably narrowed.” Early diagnosis, improved physician care, and aggressive treatment have played a major role in addressing this imbalance and improving survival.

## Pathophysiology of CFRD

**Figure 1:**
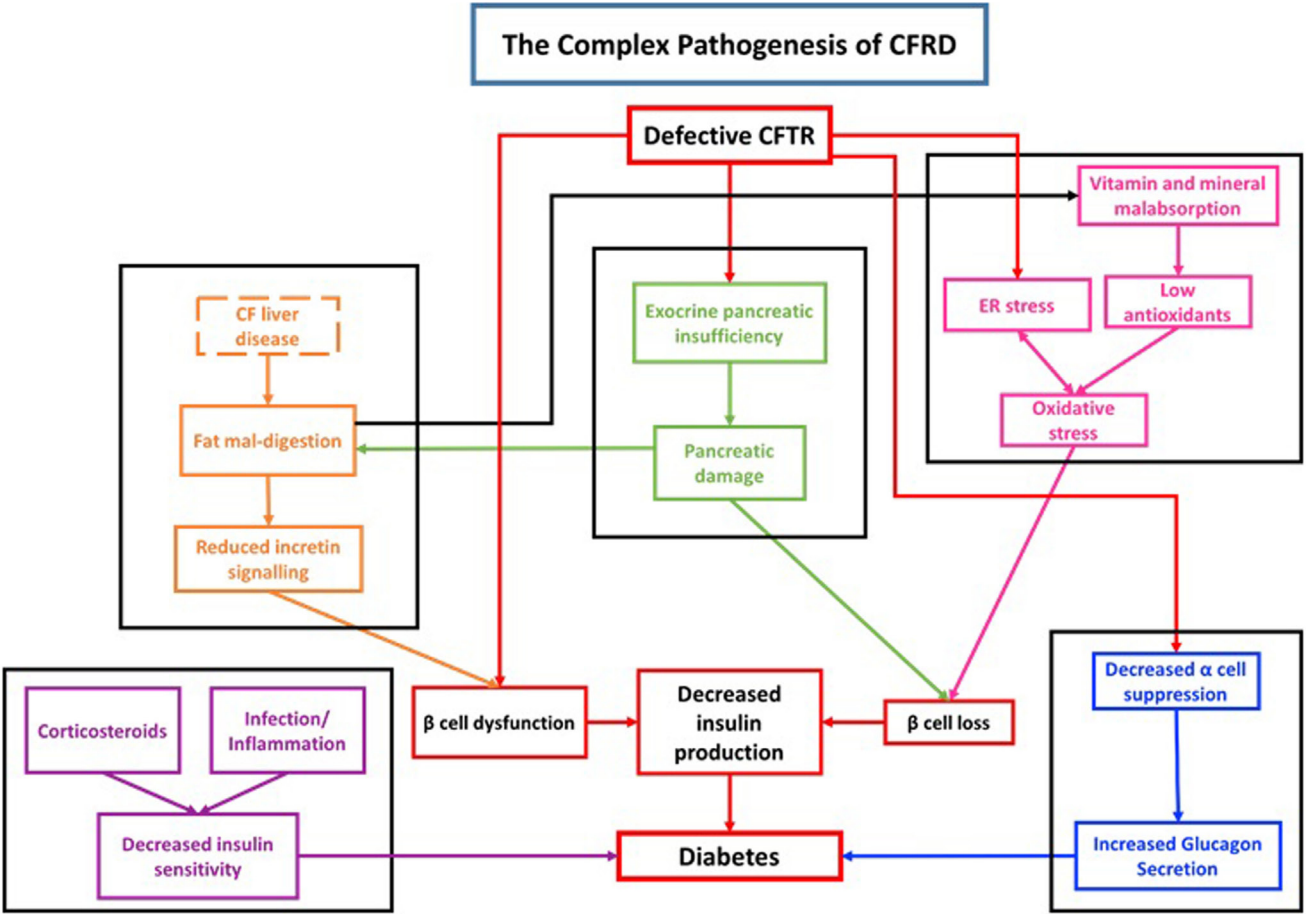
Diagram demonstrating the complex pathogenesis associated with cystic fibrosis-related diabetes (CFRD).

Although both diabetes mellitus type 1 (DM1) and CFRD are associated with a reduction in insulin production, the autoimmune pathogenesis seen in DM1 is not observed in CFRD. There is no difference in the frequency of auto-antibodies between CF patients with CFRD or without CFRD (22, 23) Thus, the pathogenesis of CFRD is distinct from DM1 and is rooted in cellular, endocrine, organ level, and system level dysfunction (Figure 1).

**Figure 2:**
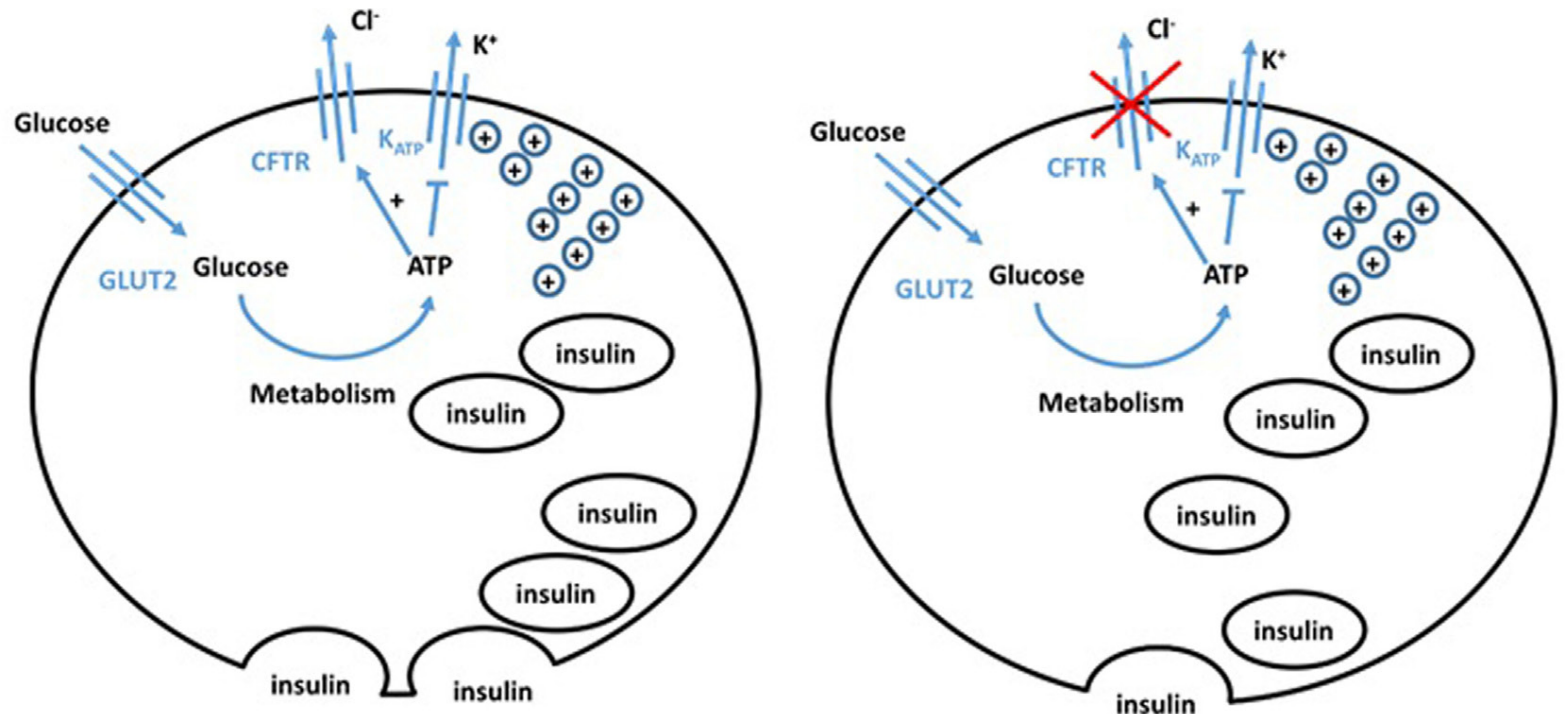
Hypothesized model of normal (left) and cystic fibrosis (CF) (right) beta cells. The defect in CFTR in the CF beta cell impairs both chloride efflux and vesicle release, impairing insulin secretion, giving an insulinopenic state.

Changes in chloride conductance have definitively been shown to alter β cell function. Using patch clamp techniques, lowered intracellular chloride levels impaired depolarization in response to glucose levels (16 mM) that normally trigger insulin release (24). CFTR is expressed in rat pancreatic α and β cells and appears to control their resting potential (Figure 2) (25). In α cells, CFTR has a role in glucagon suppression, as α cells have a KCl co-transporter which maintains a low level of chloride in the cell. The opening of CFTR, thus, induces chloride entry, causing membrane hyperpolarization, hence inhibiting glucagon secretion. CFTR dysfunction, thus, results in impaired glucagon suppression, which is observed in CFRD patients (26). Huang et al. used a CFTR mutant mouse model to explore the role of CFTR in regulating glucagon secretion (27). Their results showed that CFTR negatively regulates glucagon secretion by potentiating adenosine triphosphate-sensitive K+ (KATP) channels.

Conversely, β cells retain a high chloride electrochemical potential. CFTR opening in the β cell, therefore, allows chloride efflux and sets the cell resting potential at a level that maintains optimal insulin levels. Guo et al. (28) showed functional CFTR was indeed essential for normal β cell function. Glucose elicited membrane depolarization, calcium oscillations, and insulin secretion were abolished or reduced by inhibition or knockdown of CFTR in primary mouse β cells and β cell lines. Insulin release was also significantly attenuated in F508del mice. VX-809, a corrector of the F508del mutation, successfully rescued defects in F508del β cell lines. Similarly, Ntimbane et al. (29) showed the use of a pharmacological CFTR inhibitor gives impeded insulin release in a normal β cell line, but had little effect on CFTR knockout cells. CFTR, thus, plays an important role in β cell depolarization and insulin release. The ATP-sensitive potassium channel has previously been shown to be insufficient to cause depolarization, and therefore insulin release, on its own (30). CFTR chloride conductance is altered by ATP and, thus, CFTR may play a role in glucose-sensitive depolarization. CFTR dysfunction, therefore, gives a more hyperpolarized potential and impaired depolarization, meaning greater glucose concentrations are required for depolarization. Furthermore, CFTR dysfunction impairs the trafficking of vesicles, reducing insulin release following depolarization (7). These different experimental approaches show that hyperpolarization, impaired depolarization, and defective release of insulin vesicles by pancreatic β cells significantly contribute to the pathogenesis of CFRD. This is often overlooked by the field making it an important area for future investigation.

Cystic fibrosis-related diabetes is associated with decreased first phase insulin release (depolarization dependent), while second phase insulin (depolarization independent) remains intact (26). Knockout voltage-gated calcium channel [Ca(V)2.3(−/−)] islets have been shown to impair second phase insulin, with no impact on first phase insulin (31). The fact that second-phase insulin release is independent of depolarization and spared in CFRD is consistent with the hypothesis that CFTR is involved in β cell depolarization and first-phase insulin release. This may explain why hyperglycemia is only seen postprandially in CFRD.

A further model of why B-cell function is altered in CF is the B-cell extrinsic hypothesis. The model proposes that CFTR functions in non-cell-autonomous fashion to alter insulin secretion through paracrine or endocrine signaling and involves many different (non-B-cell) cell types. Sun X et al. used a CFTR KO model of neonatal ferret islets to show that these CFTR-expressing exocrine-derived cells affect islet insulin secretion by secreting pro-inflammatory factors such as interleukin (IL)-6 (32). This is supported by studies showing a proinflammatory state in the CF ferret pancreas at 1–2 months of age is associated with hyperglycemia and impaired β-cell function and mass (33).

These experiments demonstrate that CFTR dysfunction impairs the β cell, leading to insulinopenia. Because insulin from β cells can also exert an inhibitory effect on α cell glucagon release, this suggests a dual role of CFTR in regulating glucagon secretion; there is a direct action on α cells, and an indirect effect through insulin from β cells and both may contribute to the glucose intolerance in CF. This allows more directed management, but also highlights the importance of the future of pharmacological potentiators and correctors.

Mutations in CFTR can render β cells more susceptible to oxidative stress. β cell lines in which CFTR is silenced displayed higher levels of lipid peroxides, NF-κB signaling, and reduced antioxidant enzyme activity (SOD, catalase, and glutathione peroxidase), especially following incubation with iron/ascorbate. Decreased insulin secretion and a raised apoptosis rate in response to iron/ascorbate was also observed in CFTR silenced cells (29). Defects induced by iron/ascorbate were alleviated by the addition of Trolox, a potent inhibitor of damage by oxidative stress. However, rescue by Trolox was impaired in the CFTR silenced cell line. These *in vitro* experiments suggest that in addition to playing a role in insulin release, CFTR protects the β cells from oxidative stress.

There is evidence that oxidative stress occurs *in vivo* in CF patients. Raised levels of peroxidized fats and oxysterols are present in CF plasma, indicating abnormal lipid metabolism and increased susceptibility to oxidation of lipoprotein lipids. In addition, pancreatic insufficiency and diminished bile acid cause malabsorption of important fat-soluble antioxidants, such as carotenoids, tocopherols, and coenzyme Q-10 (34). Thus, CF patients show raised susceptibility to oxidative stress, which may affect β cells *in vivo* in the same way demonstrated *in vitro* by Ntimbane et al. (2016) (29). These effects are likely to develop over time and are reflected in gradual decline seen in glucose tolerance in CF, evidenced by the multiple glucose tolerance categories CF patients can be categorized as the expanding proportion of CF patients with CFRD as age increases.

Furthermore, pancreatic insufficiency is a hallmark of CF. The term CF was first coined to describe the disease from the appearance of the pancreas (35). Although this is not the first recorded description of CF, it placed the emphasis on the pancreas. Pancreatic insufficiency is present in 85% of CF adults, usually in those with severe mutations and plays an important role in CFRD pathogenesis (36, 37).

There is a correlation between pancreatic exocrine function and carbohydrate tolerance in CF (38, 39). Patients with pancreatic insufficiency have a low bicarbonate to chloride transport ratio (10). Bicarbonate is essential for the expansion of mucins. Impaired bicarbonate secretion, therefore, gives aggregated, poorly solubilized and less transportable mucins, which prevents fluid movement (9). CFTR dysfunction also reduces secretory volume, raising protein concentrations in the pancreatic duct, giving duct obstruction and interstitial edema (40, 41). Zymogens that fail to be secreted accumulate in the acini, where they digest pancreatic tissue (42–44). In porcine models of CF, connective tissue replaced degenerated pancreatic exocrine tissue (45). This phenomenon, where islet cells are obliterated in the midst of pancreatic exocrine tissue destruction, has been termed the “Bystander theory” (46).

Cystic fibrosis pancreatic damage begins *in utero* and, by 42 weeks post conception, pancreatic histology can distinguish between controls and CF patients (47). CF newborns have raised circulating immunoreactive trypsinogen (IRT), a CF pancreatic disease biomarker. In severe CF genotypes, this declines rapidly in the first years of life, as pancreatic damage occurs (48). Among those with severe CFTR genotypes, lowered IRT levels are associated with raised CFRD risk. The histology and immunostaining of human pancreatic islets at autopsy demonstrated changes in endocrine cell compartments (49). Whether fibrotic pancreatic damage or a pattern they termed lipoatrophic was present, the islet system was affected by a peri-insular and intra-insular sclerosis with an obvious decrease in beta cells relative to non-β cells. Together, these observations provide evidence to suggest that pancreatic exocrine insufficiency progressively causes damage to pancreatic islets, which can lead to CFRD.

Fat digestion regulates gastric emptying and postprandial glycemia *via* the entero–insular axis. Gastric emptying accounts for 34% of the variance in peak plasma glucose following a 75-g oral glucose load in normal subjects (50). CF patients suffer from impaired fat digestion (as exemplified by their steatorrhea). Pancreatic enzyme replacement therapy (PERT) improves this, but does not alter fat absorption in 20% of pancreatic insufficient CF patients (51). PERT efficiency is impaired in CF by the reduced bicarbonate levels in and amounts of pancreatic secretion, lowering intestinal pH. This delays the disintegration of the enteric coat to the distal ileum, where fat absorption is less effective (52). CFRD patients generally have more rapid gastric emptying and lower gastric inhibitory polypeptide (GIP) and glucagon-like peptide-1 (GLP-1) (which prime and stimulate insulin release) levels than CF and control groups (53, 54). Following meals, this contributes to greater postprandial glycemic excursions, consistent with CFRD being primarily associated with impaired primary insulin release. PERT normalizes GLP-1 secretion and gastric emptying, also improving (but not restoring) GIP secretion and blood glucose concentrations at 60 min (53). Thus, fat mal-digestion in CF patients contributes to the pathophysiology of CFRD and is exacerbated by any liver and gall-bladder pathology present.

## Complications of CFRD

In addition to CF complications, CFRD patients experience diabetic and additional complications, including compromised nutritional status, lung function, and susceptibility to respiratory infections. Due to the late diagnosis of CFRD, unrecognized and untreated CFRD is observed prior to firm diagnosis. Retrospective studies show in the years preceding therapy for CFRD, reductions in FEV_1_, forced vital capacity (FVC), and body mass index (BMI) are observed (55, 56). Initiating insulin therapy improves respiratory function and BMI, suggesting that the health decline observed in CFRD patients prior to diagnosis is due to reduced insulin production (55). A 4-year prospective study of NGT, impaired glucose tolerance (IGT) and CFRD patients showed overall decreases in FEV_1_ and FVC (57). Decline in respiratory function was greatest in the CFRD group, and CFRD patients in the lowest quartile of insulin production showed the greatest clinical decline. This has also been observed in a NGT CF patient group, suggesting clinical decline is correlated with low insulin production (58).

## Complications Unique to Patients with CFRD Compared to NGT CF

Excessive protein catabolism is problematic in CF lungs, which is associated with an imbalance between catabolic and anabolic enzymes (59, 60). Studies on infants (<6 months) and children (<18 years) have shown that this begins at an early age (61, 62). Adult studies have shown reduced insulin (a potent anabolic hormone) contributes to a shift toward an inflammatory catabolic state, potentially compromising lung function. Intravenous isotopic leucine has demonstrated that CF patients have significantly more systemic proteolysis than controls, that proteolysis rates correlate with glucose tolerance, and that insulin infusion suppresses systemic proteolysis (63). Despite the reduction in proteolysis and improvement seen in CFRD with insulin therapy, compared to their NGT counterparts, CFRD patients retain poorer lung function and higher mortality rates (16, 64).

The decline in pulmonary function observed in CFRD may be due to insulinopenia. The lung has recently been identified as a site of pathology in non-CF diabetic patients; however, the functional reserve in the lung means that this is not problematic for these patients. Human diabetic lungs display decreased pulmonary capillary blood volume, lung elastic recoil, FVC, and FEV_1_, while also showing a thickened basal lamina and increased collagen and elastin deposition. These effects compromise alveolar space, diffusion, and breathing efforts (65–70). Mouse models of diabetes show that diabetic and control mice produce similar levels of collagen and elastin, but diabetic mice exhibit lower total protein production. The rate of breakdown of connective tissue is lower in diabetic mice (71). Cavan et al. (72) hypothesize that the non-enzymatic glycation and crosslinking of collagen by airway glucose in the lung reduces the rate of collagen breakdown. Pulmonary changes in DM1 and DM2 are likely to be seen in CFRD, contributing to reduced lung function. Supporting this hypothesis, CT scans of CF patients showed patients with CFRD have more structural lung disease compared to NGT CF patients, with more significant airway thickening and parenchymal changes (73).

Autonomic neuropathy is present in 52% of CFRD patients, frequently resulting in diminished cardiorespiratory reflexes (74). Neuropathy may also affect the phrenic nerve, reducing lung function by affecting inspiratory muscle innervation. In addition, there is a decline in inspiratory muscle strength in DM1 patients (75). Similar changes in the skeletal musculature also occur in DM2 (76). These are likely to occur in CFRD and, therefore, affect pulmonary function, though this has not been explicitly investigated in CFRD, which needs to be addressed.

Overall, studies of lung pathology in patients with DM1 and 2 suggest distinct pathological changes may occur in the lungs of CFRD patients. These are superimposed on the pulmonary changes seen in NGT CF patients, which exacerbate pulmonary status.

As a consequence of elevated blood glucose levels (BGL), glucose can reach body compartments it is normally absent from—including the airways. Glucose becomes detectable in the airway secretions of healthy volunteers at BGLs between 6.7 and 9.7 mmol/L (77), suggesting active processes are involved in preventing glucose from entering the airways. Above threshold, airway glucose rises in a linear fashion. It was found that in CFRD patients, glucose appears in airway secretions at a BGL of 8 mmol/L, and the study participants’ BGLs were above this level for 50% of the day (78). Glucose in bronchial aspirates in non-CF intubated patients raises the risk of respiratory infection by pathogenic bacteria, including MRSA (79). CFRD is a risk factor for infection by *P. aeruginosa* (PSA), the dominant pulmonary pathogen in CF patients (80). *In vitro* studies show that PSA encourages glucose to enter the airway apical space *via* the paracellular pathway (81). A murine model of CFRD demonstrated an exaggerated, but less effective, inflammatory cell response to intratracheal PSA challenge when compared to control, CF, or diabetic mice (82). The CF and diabetic mice did show a diminished ability to control infection, however, their responses were significantly better than that of the CFRD mice. Thus, airway glucose in CFRD favors pathogenic bacterial growth and blunts the response to infection, leaving CFRD patients more susceptible to pulmonary infection.

## Diabetic Complications in CFRD

Macrovascular atherosclerotic disease is a common complication in non-CF diabetic patients but is rarely observed in CFRD (43, 83). Arterial stiffness in CF patients is increased in CFRD (43). Figueroa et al. (84), however, demonstrated that there is little correlation between abnormal lipid concentrations and glucose tolerance; instead, they found that hypertriglyceridemia in CF is related to chronic low-grade inflammation. Whether hypertriglyceridemia increases the risk of cardiovascular disease in people with CF is unknown, but as CF patients live longer, in those with family histories of cardiovascular disease, this may arise. Hypertriglyceridemia could prove to be difficult to manage as CF patients are recommended to obtain 40% of their calorific intake from fats. There have only been case reports of CFRD patients with symptomatic single and multi-vessel coronary disease thus far (85, 86).

Microvascular complications have been observed in patients with CFRD for 10 years or more (74, 87–89). A well-controlled comparison study between CFRD and DM1 patients showed no difference in the prevalence of peripheral neuropathy, nephropathy, or microvascular complications, while microalbuminuria was more common in CFRD and retinopathy was less common (88).

The prevalence of acute kidney injury in CF individuals is well documented (90) but few studies have investigated the prevalence of chronic kidney disease (CKD). A cross-sectional study by Berg et al. showed a prevalence of moderate CKD of 2.7% (11% if transplanted individuals included) in CF individuals, which is higher than in the general population of similar age (91). This supports previous studies, including a large American cohort study of 11,912 CF adults where CKD prevalence was 2.3% (92) and a British retrospective 4-year study in which prevalence was 2% (93). Thus, these observations call for further studies to investigate predictors of CKD in CF individuals and find targets for therapeutic intervention.

## Diagnosis of CFRD

The identification of CFRD is important for a number of reasons. In the 2–5 years prior to diagnosis, the pulmonary and nutritional status of CFRD patients deteriorates (55, 56). Untreated CFRD gives further decline. In addition to CF complications, CFRD also presents unique issues, such as airway glucose, which promotes bacterial lung infections. Diabetic complications also affect CFRD patients. These factors culminate in increased mortality in CFRD.

There are multiple methods available for the diagnosis of diabetes. Both fasting blood glucose and glycated hemoglobin (HbA1c) levels, which are used in diabetes mellitus (DM), are unsuitable for diagnosing CFRD. CFRD patients experience transient postprandial hyperglycemia, while fasting hyperglycemia never develops or only appears years following initial diagnosis. Solely testing fasting glycemia would have missed CFRD in 20% of patients (94).

HbA1c measures average glycemic status over 60–90 days, the lifespan of a red blood cell. CFRD patients experience transient postprandial hyperglycemia. This does not significantly affect the glycation status of red blood cells and HbA1c can be spuriously low in patients with CF, possibly due to increased red blood cell turnover due to inflammation (46). Indeed, the HbA1c test has a sensitivity of only 50% in detecting CFRD when compared to the oral glucose tolerance test (OGTT) (the gold standard) and does not correlate with mean plasma glucose levels (95, 96). For these reasons, neither fasting glucose levels nor the HbA1c test serves as effective means for diagnosing CFRD.

The American Diabetes Association recommends testing CF patients from 10 years of age using the OGTT (97). The conventional OGTT, however, has been shown to have weak capacity to diagnose DM in CF individuals (98), as the inherent variability of the test and the variability observed in individual CF patients over time, means it does not accurately reflect glucose handling (99). The test itself is inconvenient and time consuming, thus adding to the patient’s already high burden of investigations, treatments, and hospital visits, causing low adherence to annual screenings (100). Moreover, when the OGTT values were established, the cut-offs defining CFRD were taken from Pima Native Americans with Type 2 Diabetes, in which they were used to forecast microvascular complications (e.g. retionapthy) and may not be appropriate to use in CF (101).

Furthermore, there is increasing evidence that early insulin deficiency in CF has a significant impact on clinical status, prior to the 120-min OGTT diagnosis of diabetes (102). Thus, a model that predicts early insulin deficiency glucose abnormalities should be sought, which will be a more proactive and cost-effective strategy. Hence, continuous glucose monitoring (CGM) systems are an emerging technology that allows frequent (every 5 min) glucose measurements to monitor glucose trends in real time. A RCT by Bolinder et al. investigated the use of one such flash glucose sensor system (103). This resulted in a significant reduction in time and incidence of hypoglycemia, demonstrating that the technology is a safe and effective replacement to conventional self-monitoring of blood glucose. As CGM systems become more affordable, they are already being used in clinical practice, so it is feasible that this technology can be used to diagnose DM in CF individuals. Recent studies have shown that, in adult patients with CF, CGM systems identified a greater degree of impaired glucose metabolism than the gold standard 2-h OGTT. The increased frequency of monitoring glucose changes during real-life settings for 3–5 days improves the chance to detect more glycemic abnormalities during basal and postprandial conditions compared to other short-timed methods (104). However promising, their use as a diagnostic tool is still in development. More work is needed to establish a consensus on screening parameters/thresholds [e.g. number of glucose elevations are not validated in CFRD (100)] and its correlation with clinical outcomes.

## Treatment of CFRD

The treatment of CFRD must be considered with the desired outcomes in mind. In non-CF diabetic patients, the major complications to be controlled are the immediate polydipsia and polyuria as well as the long-term need to reduce micro- and macrovascular complications. CFRD, however, is treated with the primary aim of reducing airway glucose as well as pulmonary and nutritional decline (43). This is achieved *via* the blood glucose lowering and anabolic effects of insulin, which improves calorie intake, body weight, airway glucose levels, and frequency of infection. Years before diagnosis, CFRD patients experience declining pulmonary and nutritional status (105). It may be advisable to begin insulin therapy early for people with or developing CFRD, as glucose tolerance declines, especially as CFRD is the result of progressive β cell dysfunction.

According to the clinical care guidelines, the only recommended treatment for CFRD is insulin (97). As well as controlling hyperglycemic excursions, the anabolic effects of insulin have been shown to be extremely efficient in reversing the adverse effects associated with CFRD (55, 56, 63). CF patients experience gastrointestinal complications. For this reason, they are recommended to consume three meals and three snacks a day, and have a recommended daily calorie intake of 120–150% of that of the general population (83, 106). Short acting insulin provides flexibility and can be adjusted for snacks, night feeds, and the carbohydrate content of meals, although a recent review on managing CFRD found no significant difference between using long-acting insulins, short-acting insulins or oral hypoglycemic agents to control hyperglycemia in CF individuals (107). Insulin treatment carries the risk of hypoglycemia, which can occur during daily prescribed physiotherapy in CFRD. The prevalence of hypoglycemia in CFRD is comparable to that in DM1; however, episodes in CFRD patients seem to be less severe (108). The use of insulin pumps provides an alternative approach to basal/bolus insulin in treating CFRD. This has been shown to further reduce protein catabolism and hypoglycemic events in patients (109). In addition, fasting and postprandial BGLs improve, as do body weight, lean mass and HbA1C. This study was limited, however, as only nine patients were involved and comparisons were made to baseline, before use of the pump, rather than a control group. Nevertheless, insulin therapy provides huge improvements in CFRD patients.

Incretins act to prime and stimulate β cells during digestion and maximize insulin release in response to postprandial hyperglycemia. Therapies based on incretins are used in diabetics (110). Current guidelines for DM2 recommend the use of exenatide and liraglutide. Shorter acting GLP-1 agonists may be better for the treatment of early stage CFRD; these improve insulin release and slow gastric emptying, ameliorating glycemia. GLP-1 has been shown to stimulate ERK signaling in β cells and GLP-1 receptor agonists could potentially promote β cell survival (111). However, GLP-1 agonists exhibit side effects including nausea and vomiting which may require their withdrawal. Furthermore, PERT has already been shown to normalize GLP-1 production, slow gastric emptying, and improve post-prandial hyperglycemia in CF patients compared to control (46). Finally, as DPP-4 breaks down GLP-1 in the circulation, inhibitors of this enzyme have been suggested as an alternative to the use of GLP-1 agonists (44).

The use of PERT is also potentially beneficial in CFRD. Issues with fat mal-digestion exist in CFRD patients and PERT aims to improve this by slowing gastric emptying. Pancreatic insufficiency means that the pancreas is impaired in secreting of proteases, lipases and amylases, and nutrients are thus not properly digested nor absorbed. Unprotected enzymes cannot be orally administered, due to denaturation in the stomach. PERT encapsulates exogenous enzymes in an enteric coat made of a pH sensitive polyacryl acid layer, which dissolves at a pH greater than 5.5. The enzymes are, thus, protected from the acidic stomach but are released in the alkaline duodenum, meaning pancreatic amylase, lipase, and protease can be exogenously delivered. Partially digested fats have an important role to play in triggering the enterogastric reflex, reducing gastric emptying by inhibiting the vagal stimulation of the stomach and activating sympathetic neurons, which stimulates the release of hormones such as CCK. Reinstating the proper digestion of fats also restores proper gastric emptying and increases incretin production (53).

A difficulty in optimizing PERT is that dosing can vary depending on the constituents of a meal. A fat heavy meal may require more PERT capsules, to account for the greater lipid content. Furthermore, PERT depends on an alkaline duodenum, but pancreatic insufficiency and defective bicarbonate secretion may reduce pH. This means the enteric coat may not dissolve properly, reducing the efficacy of PERT. The use of proton pump inhibitors may alleviate this problem, reducing gastric acid secretion, increasing duodenal pH, and allowing the enteric coat to dissolve (106, 112). These issues demonstrate how difficult it can be for CFRD patients to manage even well targeted therapies such as PERT.

Diabetics are commonly advised to restrict their glycemic excursions by avoiding sugary foods and consuming high fiber, low fat diets (43). This conflicts with the high calorie intake and frequent snacks recommended for CF patients, who experience nutritional decline in CFRD. High-fat diets are recommended to compensate for the poor lipid absorption in this patient group. Nutritional status and weight are key determinants in predicting mortality, with good nutritional status being associated with positive outcomes (113). Providing dietary advice to CFRD patients in order to manage their diabetes and promote nutritional status will likely require a multidisciplinary team to uniquely advise each patient.

Repaglinide stimulates insulin secretion. A study involving seven CFRD patients suggested that repaglinide does have some insulinogenic effects and reduces post-prandial glucose levels, but injected insulin remained more effective than repaglinide. This may be a dosing effect and, if higher doses of repaglinide were used, this group may have shown more improvement. This drug may be especially appropriate for CFRD without fasting hyperglycemia as it has a very short half-life of roughly 1 h, making it useful in tackling short lived post-prandial excursions (114).

As mentioned above, there is a body of evidence suggesting that the lack of insulin production in CFRD patients may be directly related to defective CFTR in β cells. Lumacaftor is a CFTR corrector that rectifies folding, preventing the degradation of CFTR. This specifically targets class II mutations, including the prevalent F508del. *In vitro*, it has been shown to improve CFTR-mediated chloride transport. Ivacaftor is a potentiator, thus improving the open probability of gating (class III) or conduction defect (class IV) CFTR mutant channels. Clinical trials combining Lumacaftor and Ivacaftor have demonstrated a modest clinical benefit for F508del CF patients; but Ivacaftor alone did not provide significant improvement (44). In addition, concerns were also raised about the side effects of lumacaftor and ivacaftor combination therapy, including transient dyspnea, liver damage, and potential interactions of lumacaftor with other drugs. Two clinical trials recently reported their findings on a new corrector agent (Tezacaftor) in combination with Ivacaftor (115, 116). The combination was efficacious in improving pulmonary function in patients 12 years of age or older who had CF and were homozygous for the CFTR Phe508del mutation, with only mild side effects. These results indicate that effective CFTR modulator therapy can be beneficial in this group of patients.

## Concluding Remarks

Cystic fibrosis-related diabetes adds to difficulties in maintaining weight, pulmonary function, and susceptibility to infection seen in all CF patients. The burden of managing a second complex condition with its own distinct complications, such as retinopathy and neuropathy, compounds the problems CFRD patients need to manage. Better understanding how altered gastric emptying and fat digestion contribute to CFRD pathogenesis has allowed for the development of targeted therapies such as PERT. Existing therapies developed for diabetics, such as insulin, have been used to effect in CFRD. However, no trials have compared the efficacy of different insulin regimens, which should be addressed urgently.

Despite recent advances, our understanding of CFRD pathogenesis, pathophysiology, and optimal treatment is incomplete. New evidence has highlighted the role of CFTR in regulating the function of β cells and protecting them from oxidative stress. Further work is required to understand how these effects of CFTR contribute to CFRD and produce more directed therapies such as glutathione to reduce oxidative stress. Second, appreciating that the pathogenesis of CFRD is most likely gradual and begins *in utero* is essential to successfully avoiding the complications experienced in the years prior to diagnosis and may form the basis of starting therapy as glucose tolerance declines.

Our expanding understanding of CF more generally led to the successful development of Ivacaftor in 2012, which has already been shown to improve insulin production in CF patients. Ivacaftor and Lumacaftor are the first therapies to be used which target the defect in CF directly, rather than its symptoms. However, each quality of life adjusted year (QUALY) Ivacaftor provides costs between £335,000 and £1.2 million. NICE uses a limit of £30,000 per QALY, putting this drug out of reach for most patients (117, 118). Hence, novel pharmacological correctors, such as Tezacaftor, which is currently under FDA and EMA review, may provide huge benefit to CFRD patients, and may be more cost-effective for health systems and patients alike.

The previously poor outcomes for CFRD compared to NGT CF patients has significantly improved in recent years. This is largely due to earlier diagnosis, improved understanding of the condition and a broader range of management options, allowing for more intensive treatment. The future of CFRD, however, may prove to be less rosy. Age increases the likelihood of developing CFRD and as survival improves, the proportion of CF adults with CFRD may exceed the current 40–50% estimate. As wider CF treatment continues to improve, the gap in mortality between CFRD and NGT CF groups may widen again. Future research should focus on understanding how CFTR mutants alter β cell function directly, giving rise to CFRD and on how to pharmacologically correct this defect, rather than simply manage symptoms.

## Author Contributions

KK and RM were involved in the conception, design, drafting, revising and final approval of the article. HM was involved in drafting, revising and final approval of the article. All authors agree to be accountable for all aspects of the accuracy and integrity of the work and approve its publication.

## Conflict of Interest Statement

The authors declare that the research was conducted in the absence of any commercial or financial relationships that could be construed as a potential conflict of interest.
